# Routine ultrasound does not improve instrument placement at operative vaginal delivery: An updated systematic review and meta‐analysis

**DOI:** 10.1002/ijgo.15948

**Published:** 2024-10-04

**Authors:** Rossana Orabona, Anna Fichera, Carolina Scala, Ambrogio P. Londero, Federico Prefumo

**Affiliations:** ^1^ Division of Obstetrics and Gynecology, ASST Spedali Civili, Department of Clinical and Experimental Sciences University of Brescia Brescia Italy; ^2^ Obstetrics and Gynecology Unit IRCCS Istituto Giannina Gaslini Genova Italy; ^3^ Department of Neuroscience, Rehabilitation, Ophthalmology, Genetics, Maternal and Infant Health University of Genova Genova Italy

**Keywords:** forceps, meta‐analysis, position, systematic review, ultrasound, vacuum, ventouse

## Abstract

Routine ultrasound before operative vaginal delivery does not improve the accuracy of instrument placement.

Two previous systematic reviews and meta‐analyses demonstrated that ultrasound assessment in addition to standard vaginal examination prior to operative vaginal delivery improves the accuracy in the diagnosis of fetal head position. However, this does not result in improved clinical outcomes.^1^, ^2^ It is not clear from previous research which step(s) of operative delivery are or are not improved by the use of ultrasound.

The aim of this updated systematic review and meta‐analysis of randomized controlled trials was to assess at which step(s) in the procedure of operative vaginal delivery ultrasound fails to improve outcomes, to provide a framework for future research. The review was performed following an a‐priori protocol (www.crd.york.ac.uk/prospero/display_record.php?ID=CRD42021261144) and is reported following the Preferred Reporting Item for Systematic Reviews and Meta‐analyses (PRISMA) statement and checklist. The study was registered with the PROSPERO database (registration number CRD42021261144). We included randomized controlled trials comparing delivery outcomes with ultrasound assessment in addition to standard vaginal examination versus vaginal examination alone prior to operative vaginal delivery.

We conducted electronic searches for eligible studies in MEDLINE, Scopus, and www.clinicaltrials.gov from inception to November 6, 2023. The full search strategies are available in Table S1. No language restrictions were used. Two trained reviewers (R.O. and F.P.) independently screened titles and abstracts for relevance. In case of disagreement, consensus was reached by discussion. If necessary, a third researcher was consulted. The full text of relevant articles was evaluated independently, and agreement about potential eligibility was reached by consensus. Again, in cases of disagreement, a consensus in inclusion or exclusion was reached by discussion, eventually consulting a third researcher.

We extracted information relating to results according to sequential step(s) in the procedure of operative vaginal delivery.^3^ A data collection sheet was designed, and RO and FP independently extracted data from eligible studies. Any discrepancies in the extracted data were resolved through discussion. Data were entered into RevMan 5.4.1 software (Review Manager 2014).

We assessed risk of bias in the included studies using the Cochrane ‘Risk of bias’ tool for randomized trials (RoB 2.0). Two review authors independently applied the tool to each included study and recorded supporting information and justifications for judgments of risk of bias for each domain (low, high, and some concerns). Meta‐analyses using a random‐effect model were used to analyze the data, and results were reported as relative risks with their 95% confidence intervals (CI).

Following screening, four trials met the inclusion criteria,^4^, ^5^, ^6^, ^7^, ^8^ covering 1007 subjects; a flow diagram of the selection process is shown in Figure S1. Study characteristics are provided in Table S2. Some outcomes of the trial by Ramphul et al.^6^ were reported in detail in the subanalysis by Ramphul et al.^6^ The effects of ultrasound assessment before operative vaginal delivery are shown in Table 1. Although ultrasound improved accuracy in diagnosis of fetal head position, it had no significant effect on the next procedural assessment, which was instrument distance from the flexion point, as assessed at the end of delivery by observation of the chignon or forceps marks. Ultrasound use was not significantly associated with any improvement in the subsequent procedural outcomes.

**TABLE 1:** Summary of outcomes analyzed in the meta‐analysis.

Outcome	Studies	Participants	Risk ratio [95% confidence interval]
Accurate diagnosis of fetal head position	2	735	1.17 [1.04, 1.31]
Instrument distance from flexion point	3	749	−0.18 [−0.43, 0.07]
Failure of first choice operative vaginal delivery	4	977	1.12 [0.76, 1.64]
Sequential instrument use	4	987	1.13 [0.71, 1.80]
Caesarean section	4	1007	0.86 [0.29, 2.51]
Shoulder dystocia	4	957	0.95 [0.54, 1.68]
5‐min Apgar score <7	4	1007	0.71 [0.19, 2.73]
Arterial pH <7	3	837	1.00 [0.45, 2.25]
Newborn trauma	4	1007	1.01 [0.58, 1.75]
Perineal trauma	4	1007	0.89 [0.54, 1.47]
Postpartum hemorrhage	3	957	1.17 [0.85, 1.60]

Systematic review and meta‐analysis were employed to summarize evidence about the effects of intrapartum sonographic evaluation of the fetal head position on the success of operative vaginal delivery steps. Most women have spontaneous vaginal births, but some women need assistance in the second stage with delivery of the baby, using either obstetric forceps or vacuum extraction. Errors in the assessment of the fetal head position might result in a failure of operative delivery with an increased risk of maternal and neonatal morbidity. Our analysis confirmed that the combination of digital and sonographic assessment before instrumental vaginal delivery is more accurate in the diagnosis of the fetal head position than the vaginal exploration alone in this setting.^1^, ^2^ However, we observed that such improved knowledge did not affect the accuracy in instrument placement, as measured by instrument mark distance from the flexion point. This observation identifies a gap in research, suggesting that the introduction of ultrasound assessment before operative vaginal delivery should be accompanied by specific training.

## AUTHOR CONTRIBUTIONS

RO and FP conceived and designed this study. RO, CS and FP contributed substantially to the acquisition of the data. RO, APL, and FP performed statistical analyses. RO, AF, CS, APL, and FP contributed to the interpretation of the results. RO and FP drafted the paper. RO, AF, CS, APL, and FP revised and approved the final version of the manuscript.

## CONFLICT OF INTEREST STATEMENT

The authors have no conflict of interest to declare.

## INFORMED CONSENT

No patient consent was necessary for this study.

## Supporting information


Figure S1. Flow diagram of the study selection process.

Table S1. Search strategies.

Table S2. Characteristics of the studies included in the systematic review.

## Data Availability

Data sharing is not applicable to this article as no new data were created or analyzed in this study.
