# Supplementary material for: Routine ultrasound does not improve instrument placement at operative vaginal delivery: An updated systematic review and meta‐analysis
Source: Int J Gynaecol Obstet. 2024 Oct 4;168(3):1335–6. doi: 10.1002/ijgo.15948 (PMC11823359; doi:10.1002/ijgo.15948)
Supplement: Supplementary file 1 — Figure S1. Flow diagram of the study selection process. [file IJGO-168-1335-s002.docx]

**Supplementary Figure 1.** Flow diagram of the study selection process.

**Identification of studies via databases and registers**

Records removed *before screening*:

Duplicate records removed (n = 3)

Records identified from:

Databases (n = 3)

Registers (n = 1)

**Identification**

Records screened

(n = 307)

Records excluded

(n = 296)

Reports sought for retrieval

(n = 11)

Reports not retrieved

(n = 0)

**Screening**

Reports excluded (n=6):

| Study | Digital object identifier | Reason for exclusion |
| --- | --- | --- |
| Ramphul 2012 | 10.1016/j.ejogrb.2012.07.029 | Survey |
| Murphy 2012 | 10.1186/1471-2393-12-95 | Study protocol |
| Popowski 2015 | 10.1002/uog.14785 | Operative vaginal delivery not an inclusion criterion |
| Masturzo 2017 | 10.1002/jcu.22477 | Operative vaginal delivery not an inclusion criterion |
| Bellussi 2018 | 10.1002/uog.19044 | Operative vaginal delivery not an inclusion criterion |
| Haumonte 2022 | 10.1016/j.ajog.2022.04.018 | Operative vaginal delivery not an inclusion criterion |

Reports assessed for eligibility

(n = 11)

Studies included in review

(n = 4)

Reports of included studies

(n = 5)

**Included**
