# Supplementary material for: Routine ultrasound does not improve instrument placement at operative vaginal delivery: An updated systematic review and meta‐analysis
Source: Int J Gynaecol Obstet. 2024 Oct 4;168(3):1335–6. doi: 10.1002/ijgo.15948 (PMC11823359; doi:10.1002/ijgo.15948)
Supplement: Supplementary file 2 — Table S1. Search strategies. [file IJGO-168-1335-s003.docx]

**Supplementary Table 1.** Search strategies.

Medline

"Randomized Controlled Trial"[PT] AND "Delivery, Obstetric"[Mesh] AND "Ultrasonography"[Mesh]

Scopus

(ALL(“operative vaginal delivery”) OR ALL(“'forceps delivery”) OR ALL(“'vacuum extraction”)) AND ALL(“ultrasound”) AND TITLE-ABS-KEY(“randomized controlled trial”)

Clinicaltrials.gov search

'Obstetric Labor Complications' AND 'Ultrasound'

Filter ‘Interventional’
