# Supplementary material for: Routine ultrasound does not improve instrument placement at operative vaginal delivery: An updated systematic review and meta‐analysis
Source: Int J Gynaecol Obstet. 2024 Oct 4;168(3):1335–6. doi: 10.1002/ijgo.15948 (PMC11823359; doi:10.1002/ijgo.15948)
Supplement: Supplementary file 3 — Table S2. Characteristics of the studies included in the systematic review. [file IJGO-168-1335-s001.docx]

**Supplementary Table 2.** Characteristics of the studies included in the systematic review.

| **First author/Year/DOI** | Study design | **n** | **Intervention** | Primary outcome measured | Risk of bias | | | | |
| --- | --- | --- | --- | --- | --- | --- | --- | --- | --- |
|  |  |  |  |  | **Randomization process** | **Deviations from intended**  **interventions** | **Missing outcome data** | **Measurement of the outcome** | **Selection of the**  **reported result** |
| Wong 2007  10.1016/j.ijgo.2007.05.021 | Single centre randomized study | 50 | Transabdominal ultrasound evaluation of fetal head position before instrumental delivery | Accuracy of vacuum cup placement | Low | Low | Low | Low | Some concerns |
| Ramphul 2014  10.1111/1471-0528.12810 | Multicentre two-arm randomized controlled trial | 514 | Transabdominal ultrasound evaluation of fetal head position before instrumental delivery | Incorrect diagnosis of fetal head position | Low | Low | Low | Low | Low |
| Ramphul 2015  10.1111/1471-0528.13186 | Observational study nested within Ramphul 2014 | 478 | Transabdominal ultrasound evaluation of fetal head position before instrumental delivery | Not applicable |  |  |  |  |  |
| Ghi 2018  10.1002/uog.19091 | Multicentre randomized controlled trial | 221 | Transabdominal ultrasound evaluation of fetal head position before instrumental delivery | Incidence of failed vacuum extraction and need to perform emergency Cesarean section | Low | Low | Low | Low | Some concerns |
| Barros 2021  10.1111/aogs.14065 | Multicentre randomized controlled trial | 222 | Transabdominal ultrasound evaluation of fetal head position + transperineal evaluation of the angle of progression before instrumental delivery | Composite maternal and neonatal morbidity | Low | Low | Low | Low | Some concerns |
